# Glutamine Improves Oxidative Stress through the Wnt3a/*β*-Catenin Signaling Pathway in Alzheimer's Disease In Vitro and In Vivo

**DOI:** 10.1155/2019/4690280

**Published:** 2019-04-16

**Authors:** Yuan Wang, Qiang Wang, Jie Li, Gang Lu, Zhibin Liu

**Affiliations:** ^1^College of Acumoxibustion and Massage, Shaanxi University of Chinese Medicine, Xianyang 712046, China; ^2^Shaanxi Key Laboratory of Acupuncture and Medicine, Xianyang 712046, China

## Abstract

**Background/Aims:**

Alzheimer's disease (AD) is the most common neurodegenerative disease, and all researchers working in this field agree that oxidative stress is intimately associated with Alzheimer disease. In this study, we hypothesized that glutamine (Gln) offers protection against oxidative stress injury in SAMP8 mice as well as the underlying mechanism.

**Methods:**

The SAMP8 mice received glutamine intragastrically for 8 consecutive weeks to evaluate the protective effect of glutamine on oxidative stress in AD mice involving Wnt3a/*β*-catenin signaling pathway. In addition, rat pheochromocytoma tumor cell line PC12 was pretreated with 32 *μ*M glutamine for 2 h followed by 24 h incubation with 40 *μ*M A*β*25-35 to obtain in vitro data.

**Results:**

In vivo the administration of glutamine was found to ameliorate behavioral deficits and neuron damage, increase superoxide dismutase (SOD) and glutathione peroxidase (GSH-XP) activity, reduce the malondialdehyde (MDA) content, and activate the Wnt3a/*β*-catenin signaling pathway in SAMP8 mice. In vitro glutamine treatment decreased the toxicity of A*β*25-35 on PC12 cells and prevented apoptosis. Additionally, glutamine treatment increased SOD and GSH-XP activity and decreased MDA content and increased Wnt3a and *β*-catenin protein levels. Interestingly, the DKK-1 (Wnt3a/*β*-catenin pathway inhibitor) decreased the antioxidant capacity of glutamine in A*β*25-35-treated PC12 cells.

**Conclusion:**

This study suggests that glutamine could protect against oxidative stress-induced injury in AD mice via the Wnt3a/*β*-catenin signaling pathway.

## 1. Introduction

Alzheimer's disease (AD), also known as senile dementia, is a chronic neurodegenerative disorder characterized by progressive cognitive impairment and behavioral damage [1]. It is predicted that the number of AD patients will rise to 90 million in 2050 worldwide [2]. More worrying is that China's aging population is increasing by more than 8 million every year, and the number of AD patients in China is expected to be the sum of all developed countries in 2040 [3]. AD has become the fourth leading cause of death in humans after cardiovascular disease, cancer, and stroke. Amyloid cascade is an important hypothesis in the pathogenesis of AD. Aggregation of Amyloid *β*-protein (A*β*) induces an oxidative stress response that damages mitochondria. Oxidative stress, in turn, further promotes the aggregation of A*β* and the phosphorylation of tau, a microtubule-related protein, aggravating the imbalance of REDOX reactions in the brain of AD patients. Oxidative stress is a common key point connected with various pathogenic mechanisms [4]. Therefore, finding suitable antioxidants for appropriate clinical intervention is an effective means to prevent AD.

Glutamine (Gln) is the most abundant-free amino acid in plasma, acting on oxygen-free radicals and playing an important role in vascular disease [5], diabetes [6], neurodegenerative diseases [7, 8], and various cancers. As the main energy supply substance for mitochondria to form ATP, the oxidation of Gln can eliminate some strong oxidizing substances to protect some important components of cells from oxidative damage [9]. The intracellular and extracellular glutamine is essential for neuronal health. A previous research pointed out that the dietary supplementation of glutamine has significant neuroprotective effects and help restore homeostatic functions that are lost in AD [10]. However, whether the glutamine can affect the level of oxidative stress to play a neuroprotective effects in AD mice model is not clear.

As a key molecular pathway, the Wnt3a/*β*-catenin signaling pathway regulates neuronal survival, differentiation, axonal extension, neurogenesis, synapse formation and plasticity, and neuroprotection [11]. Previously, the pathway was related to research on the treatment of Parkinson's disease [12]. Increasing evidence showed that this pathway participated in the neuronal differentiation and apoptosis in Alzheimer's disease model [13, 14]. Wnt3a/*β*-catenin signaling is also critically associated with the occurrence and development of oxidative stress [15]. Thus, our study aimed to evaluate whether glutamine protects against oxidative stress-induced injury via activation of the Wnt3a/*β*-catenin signaling pathway in a mouse model of AD.

## 2. Materials and Methods

### 2.1. Reagents

A*β*25-35 [Amyloid beta-peptide (25-35)] was purchased from MCE (HY-P0128; NJ, USA) and the purity of A*β*25-35 was ≥ 98%. DKK-1 (Wnt3a/*β*-catenin pathway inhibitor; SRP3258) and glutamine (Gln; 1294808) were purchased from Sigma-Aldrich (Merck KGaA, Darmstadt, Germany). Additional reagents employed in the present study were commercially available and of analytical purity.

### 2.2. Animals and Grouping

Thirty male senescence accelerated mouse SAMP8 (20~30 g, three-month-old) and ten normal aging control mouse SAMR1 were purchased from the animal center of the West China Medical College of Sichuan University, Chengdu, China. All animals were housed in the animal laboratory under controlled, conventional conditions (temperatures of 24±1°C, relative humidity of 60±10%, and 12 h light-dark cycle) and were allowed free access to food and water during the experimental session. Following a week of adaptation, mice were randomly divided into four groups (n=10): the control (SAMR1), model (SAMP8), glutamine-low (250 mg/kg), and glutamine-high (500 mg/kg) groups. After grouping, the mice were immediately administered treatments. The mice in the control and sham groups received 1 mL saline by intragastric administration, and the mice in the glutamine groups received an equal volume of 250 mg/kg and 500 mg/kg glutamine intragastrically. The treatment was given once a day for 8 consecutive weeks.

### 2.3. Behavioral Assessment

A step-down passive avoidance test was performed to detect the learning and memory of mice [16]. The platform reaction box (10x10x5 cm) was divided into two sections by a copper gate with continuous electrical stimulation (36 V) at the bottom of the box. A 4.5 cm inner diameter and height rubber pad was placed at the right rear corner of each box to serve as a safe area for mice to avoid electric shock. In the training session the animals were placed on the platform for 3 min. The time taken to react to jump to the pad (reaction time) and the number of electric shocks they received within 5 min (error frequency) were recorded as learning achievements. After 24 h, the animals were again placed into the platform for 3 min and then set on the pad. The first time they jumped off the pad (latent period) and the number of electric shocks they received within 5 min (error frequency) were recorded as memory achievements.

### 2.4. HE Staining and TUNEL Staining

Five mice in each group were anesthetized by an intraperitoneal injection of 3% pentobarbital sodium (50 mg/kg); brain tissues were taken out immediately and fixed with 4% paraformaldehyde for 48 ~ 72 h. The hippocampus was embedded in paraffin, and 5-*μ*m-thick paraffin sections were prepared. The pathological changes of tissues were observed under light microscope following hematoxylin and eosin (HE) staining. The apoptotic cells were detected using a TUNEL assay kit (T2190; Beijing Solarbio Science & Technology Co., Ltd., Beijing, China). The apoptotic cells exhibited brown staining within the nucleus. Images were captured with a fluorescence microscope (Olympus Corporation, Tokyo, Japan).

### 2.5. Detection of Superoxide Dismutase (SOD), Glutathione Peroxidase (GSH-Px), and Malondialdehyde (MDA) Contents

After the intraperitoneal injection of 3% pentobarbital sodium (50 mg/kg) for anesthesia, brain tissue was removed immediately, placed on an ice tray to isolate hippocampus tissues, and weighed on an accurate electronic scale. Then, the tissues were shredded using an ophthalmic scissor and normal saline was added (1:10) to produce 10% brain tissue homogenate. The homogenate was centrifuged at 4°C for 10 min, obtaining the supernatant for future use. SOD, GSH-Px activity, and MDA contents were detected by the colorimetry method using a microplate reader according to the kit instructions (SOD: A001-1-1; GSH-Px: A005; MDA: A003-1; Nanjing Jiancheng Bioengineering Institute, Jiangsu, China).

Cells from each group were collected and digested with trypsin. Following the cells were disrupted at 4°C by an ultrasonic cell disruptor and the lysate was centrifuged at 1000 r/min at 4°C for 10 min. A total of 100 *μ*l supernatant was obtained to detect the OD values using a microplate reader according to the instructions of SOD, GSH-Px, and MDA kit, and the content was calculated.

### 2.6. Western Blot Assay

Protein samples were prepared from brain tissues and PC12 cells using RIPA lysis buffer (AR0105; Boster, Wuhan, China) and quantified with a Protein Assay kit (AR0146; Boster). In order to detect the levels of protein expression, protein samples were separated by 10% SDS-PAGE gel and then transferred onto a PVDF membrane (C3117; Millipore, MA, USA). Following sealed with 5% skimmed milk powder at room temperature for 1 h, the membranes were incubated with rabbit anti-Wnt3a (#2721), rabbit anti-*β*-catenin (#8480), and rabbit anti-*β*-actin (#4970; 1:1000; Cell Signaling Technology, MA, USA) at 4°C overnight and then incubated with goat anti-rabbit IgG at room temperature for 1 h. *β*-actin was used as inner loading control. Protein bands were visualized using an ECL chemiluminescence kit (WBULS0500; EMD Millipore).

### 2.7. Cell Culture and Proliferation Assay

Cells of the rat pheochromocytoma tumor cell line PC12 were purchased from Procell Life Science & Technology Co., Ltd (CL-0412; Wuhan, China). PC12 cells were cultured in RPMI-1640 medium (PM150115; without glutamine; Procell Life Science & Technology Co., Ltd) supplemented with 10% Fetal Bovine Serum (10099-141; FBS; Gibco, CA, USA) and incubated at 37°C in 5% CO_2_.

Cell proliferation was measured using a Cell Counting Kit-8 (CK04; CCK-8; Dojindo, Kumamoto, Japan) assay. Cells in log phase were collected and seeded into 96-well plates at a density of 6×10^3^/well. Then cells were cultured in 10% CCK-8 for 1 h and the absorbance value was measured at 450 nm using a microplate reader (Thermo Fisher Scientific, MA, USA).

### 2.8. Annexin-V/Propidium Iodide (PI) Double-Staining and Flow Cytometry Assays

Following washing, trypsin digestion, and centrifugation, PC12 cells were resuspended in 100 *µ*l binding buffer (1x10^5^ cells) with 5 *µ*l Annexin V-FITC and 5 *µ*l PI (CA1020; Beijing Solarbio Science & Technology Co., Ltd.) for 15 min in the dark. Subsequently, cell apoptosis was detected using a FACSCaliburTM Flow Cytometer (BD Biosciences) within 1 h.

### 2.9. Statistical Analysis

Statistical analysis was performed using SPSS20.0 software (IBM Corp., Armonk, NY, USA). Values were presented as the means ± standard deviation (SD) from three separate experiments. Differences among multiple groups were compared by one-way analysis of variance (ANOVA) with Dunnett's post-tests or two-way ANOVA with Bonferroni's post-tests. The differences were considered statistically significant at p < 0.05 and p < 0.01.

## 3. Results

### 3.1. Glutamine Enhances Learning and Memory Abilities of SAMP8 Mice

A passive avoidance apparatus was performed to examine the learning and memory abilities of SAMP8 mice following glutamine treatment. As shown in Table 1, compared with the control group, with respect to learning ability, the reaction time and error time in the model and glutamine-low groups were significantly increased, respectively. Compared with model group, the Gln-high group displayed reduced reaction time and error time. With respect to memory ability, the reaction time in the model and glutamine-low groups was significantly decreased compared to control group; the reaction time in the Gln-high group was significantly increased compared to model group. These results indicated that the high concentration of glutamine could enhance the learning and memory ability of SAMP8 mice.

### 3.2. Glutamine Alleviates Neuron Damage in SAMP8 Mice

**Figure 1:**
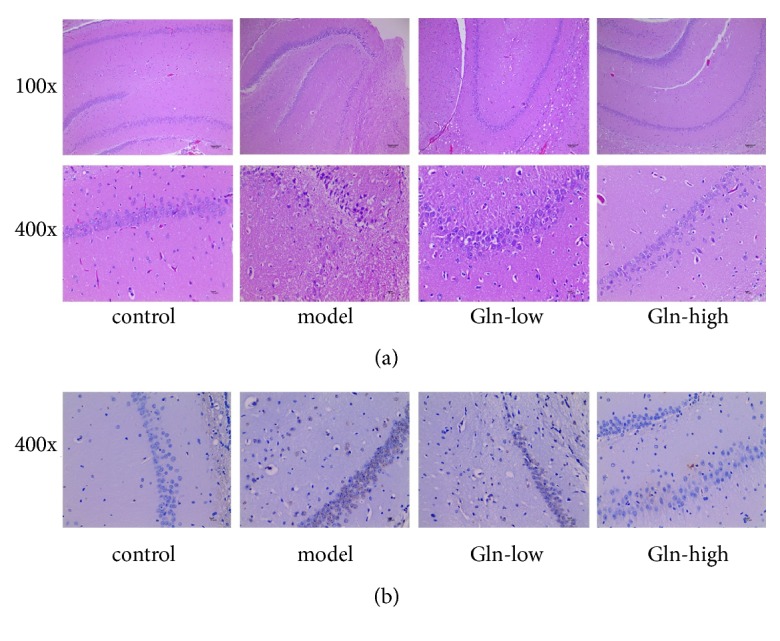
Effects of glutamine on the damage of hippocampus neurons in SAMP8 mice. (a) Images of brain tissue of mice following hematoxylin-eosin staining. (b) Images of brain tissue following TUNEL staining; nucleus of apoptotic cells were stained brown.

Morphological changes in the hippocampus were measured with H&E staining (Figure 1(a)). Compared with the control and Gln-high groups, the model and Gln-low groups had sparsely and disorderly arranged cells, substantial reduced pyramidal cells, nucleus pycnosis observed, and nucleus stained with deep blue. Apoptotic cells were detected by TUNEL staining (Figure 1(b)). The TUNEL-positive cells were observed in the model and Gln-low groups, and almost no TUNEL-positive cells were observed in the control and Gln-high groups. These results indicated that the high concentration of glutamine could improve the abnormal structure and apoptosis of the hippocampus cells in SAMP8 mice and could improve the damage of hippocampus neurons.

### 3.3. Glutamine Strengthens the Antioxidant Capacity in SAMP8 Mice

**Figure 2:**
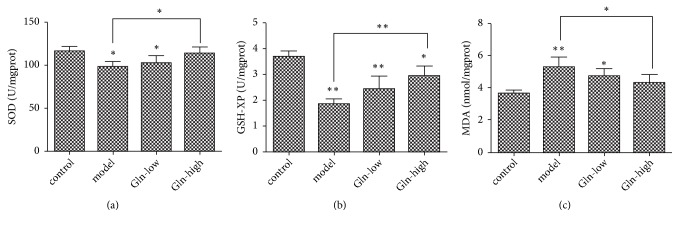
Effect of glutamine on activity of SOD and GSH-XP and content of MDA in hippocampus of SAMP8 mice. (a) SOD activity in each group. (b) GSH-XP activity in each group. (c) MDA content in each group. Data were obtained from three independent experiments. The results were presented as the mean ± standard deviation. ^*∗*^p < 0.05 and ^*∗∗*^p < 0.01.

As shown in Figure 2, compared with control group, the activity of SOD and GSH-XP in hippocampus of the model and Gln-low groups was significantly decreased, where the content of MDA was markedly increased. Compared with model group, the Gln-high group displayed reduced MDA content, increased SOD, and GSH-XP activity. These results showed that the high concentration of glutamine could improve antioxidant capacity of SAMP8 mice.

### 3.4. Glutamine Treatment Activates the Wnt3a/*β*-Catenin Signaling Pathway in SAMP8 Mice

**Figure 3:**
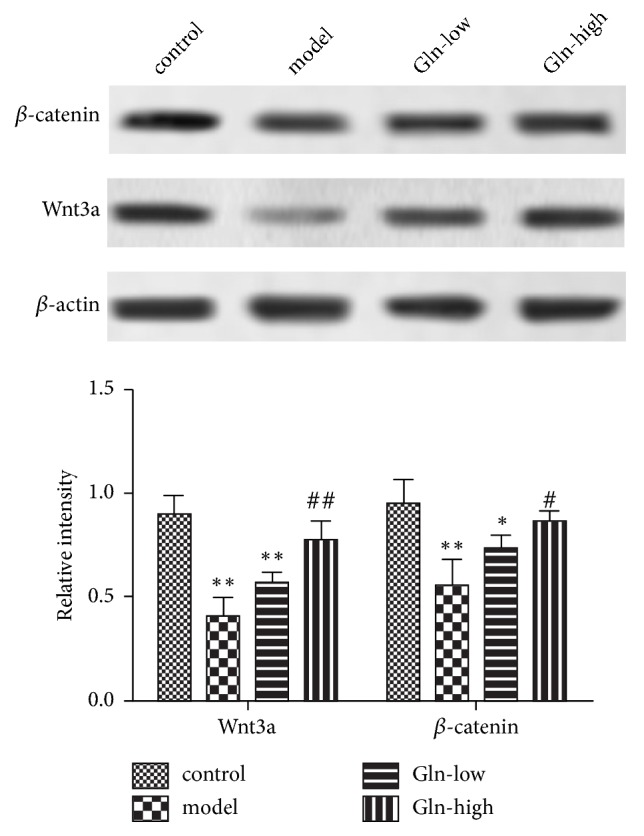
Wnt3a and *β*-catenin protein expression levels in hippocampus of SAMP8 mice following pretreatment with glutamine. The expression levels of Wnt3a and *β*-catenin protein were examined by western blotting. The experiments were repeated three times. The results were presented as the mean ± standard deviation. ^*∗*^p < 0.05 and ^*∗∗*^p < 0.01, compared with control group. ^#^p < 0.05 and ^##^p < 0.01, compared with model group.

The western blot result indicated that, compared with control group, the expression levels of Wnt3a and *β*-catenin in hippocampus of SAMP8 mice were significantly decreased. Compared with model group, the Wnt3a and *β*-catenin protein levels were significantly increased in Gln-high group (Figure 3). These results indicated that the Wnt3a/*β*-catenin signaling pathway was inhibited in SAMP8 mice, and the high concentration of glutamine could reverse.

### 3.5. The Cytotoxicity of A*β*25-35 on PC12 Cells

**Figure 4:**
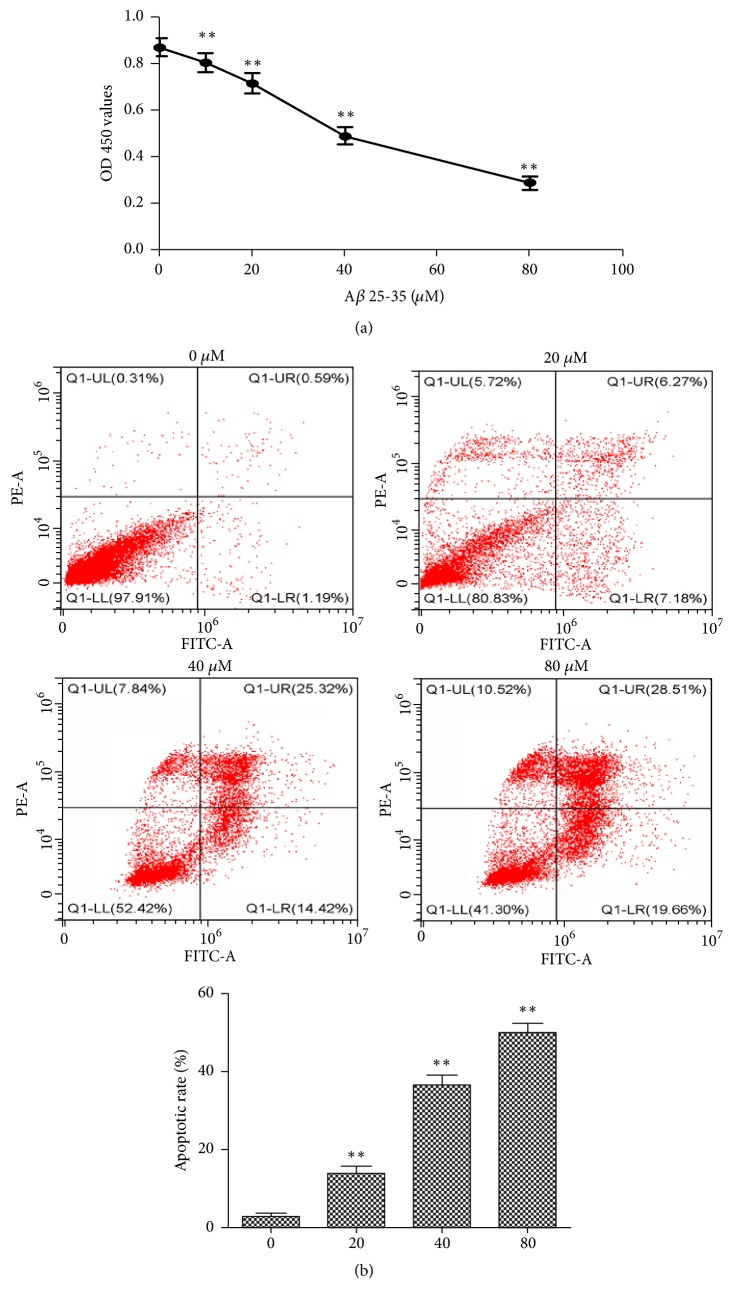
Effects of A*β*25-35 on the proliferation and apoptosis of PC12 cells. (a) PC12 cells were incubated with A*β*25-35 (0, 10, 20, 40, and 80 *µ*M) for 24 h. Cell viability was determined by a Cell Counting Kit-8 assay. (b) Cell apoptosis was detected by flow cytometry following treatment with A*β*25-35 (20, 40, and 80 *µ*M) for 24 h. Data was obtained from three independent experiments. The results were presented as the mean ± standard deviation. ^*∗∗*^P < 0.01.

To simulate pathological damage of AD in vitro, PC12 cells were treated with different concentrations of A*β*25-35 for 24 h, and the inhibition of cell proliferation was detected via a CCK-8 assay. As shown in Figure 4(a), the cell proliferation was significantly decreased in a dose- dependent manner, with IC50 value of 41.601 *μ*M. Thus, we chose 40 *µ*M and 24 h as the concentration and time of the follow-up study. To determine whether the cytotoxicity of A*β*25-35 against PC12 cells induces apoptosis, the present study analyzed apoptotic rate by flow cytometry following 24 h of treatment with A*β*25-35. As shown in Figure 4(b), the apoptotic rate of PC12 cells was significantly increased in a dose-dependent manner. These results indicated that the PC12 cells damage can be induced by A*β*25-35 and can be used in the establishment of AD cell model.

### 3.6. Glutamine Suppressed Cytotoxicity Induced by A*β*25-35 in PC12 Cells

**Figure 5:**
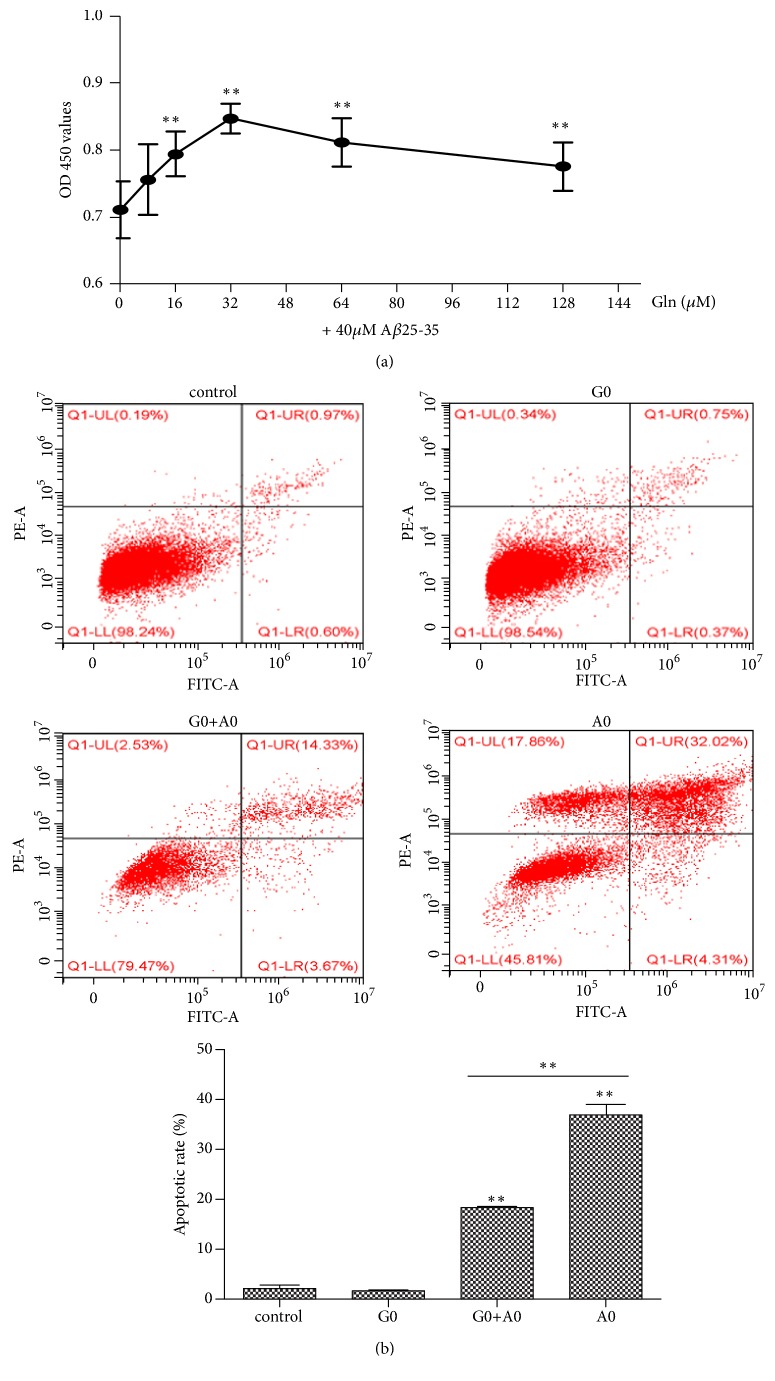
Protective effect of glutamine on PC12 cell injury induced by A*β*25-35. (a) PC12 cells were incubated with 40 *μ*M A*β*25-35 for 24 h following pretreatment with glutamine (0, 8, 16, 32, 64, and 128 *µ*M) for 2 h. Cell viability was determined by a Cell Counting Kit-8 assay. (b) PC12 cells were pretreated with glutamine (32 *µ*M) for 2 h, followed by incubation with or without A*β*25-35 (40 *μ*M) for 24 h. Cell apoptosis was detected by flow cytometry following. Data was obtained from three independent experiments. The results were presented as the mean ± standard deviation. ^*∗∗*^P < 0.01. 32 *μ*M of glutamine showed as G0, and 40 *μ*M of A*β*25-35 showed as A0.

To investigate whether the glutamine plays a protective role on A*β*25-35 induced PC12 cell injury, cells were pretreated with different concentrations of glutamine for 2 h and then incubated with 40 *μ*M A*β*25-35 for 24 h, followed by observation of PC12 cell proliferation and apoptosis. As shown in Figure 5(a), the cell proliferation was significantly increased following glutamine pretreatment, and the cell proliferation was the highest when glutamine concentration was 32 *µ*M. The flow cytometry results showed that the apoptotic rate of PC12 cells in the G0+A0 group was significantly lower than that in the A0 group (Figure 5(b)). These results suggested that the glutamine protected PC12 cells from damage induced by A*β*25-35.

### 3.7. Glutamine Treatment Activates the Wnt3a/*β*-Catenin Signaling Pathway in AD Cell Model

**Figure 6:**
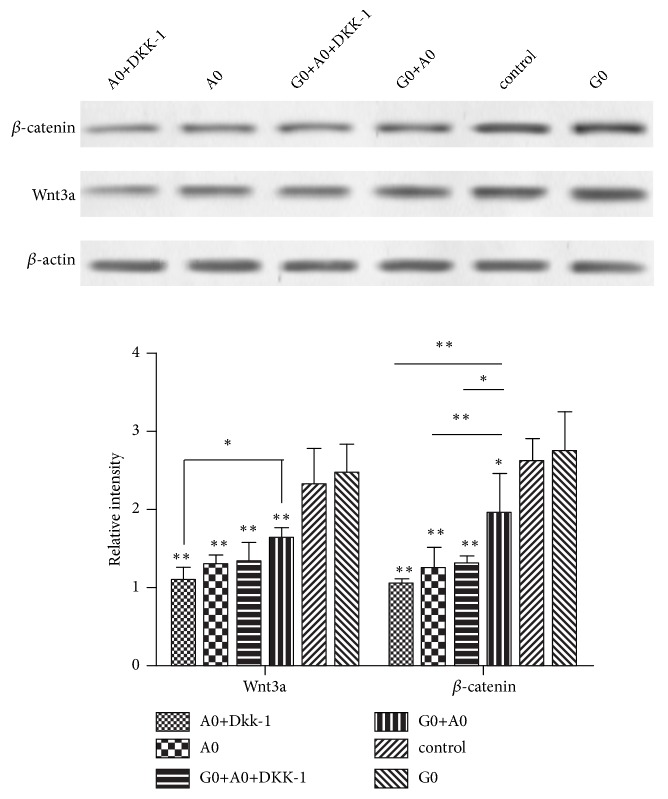
Wnt3a and *β*-catenin protein expression levels in AD cell model following pretreatment with glutamine and DKK-1. The expression levels of Wnt3a and *β*-catenin protein were examined by western blotting. The experiments were repeated three times. The results were presented as the mean ± standard deviation. ^*∗*^p < 0.05 and ^*∗∗*^p < 0.01.

To elucidate whether the protective effect of glutamine on PC12 cell damage induced by A*β*25-35 is through activation of the Wn3a/*β*-catenin signaling pathway, western blot assay was performed to detect the expression of Wn3a and *β*-catenin proteins. As shown in Figure 6, the expression of Wnt3a and *β*-catenin protein was decreased in different degrees in the remaining groups compared with the control group, indicating that the Wnt3a/*β*-catenin pathway was inhibited in AD cell model. Compared with the A0 group, the expression of *β*-catenin protein was significantly increased in the G0+A0 group, indicating that glutamine pretreatment of cells can alleviate the inhibition of Wnt3a/*β*-catenin pathway. Compared with the G0+A0 group, the Wnt3a and *β*-catenin proteins in the A_0_+DKK-1 and G_0_+A_0_+DKK-1 groups were significantly decreased, indicating that the DKK-1 can inhibit the activation of Wnt3a/*β*-catenin pathway.

### 3.8. The Blocking of Wnt3a/*β*-Catenin Signaling Pathway Decreased the Antioxidant Capacity of Glutamine in PC12 Cells

**Figure 7:**
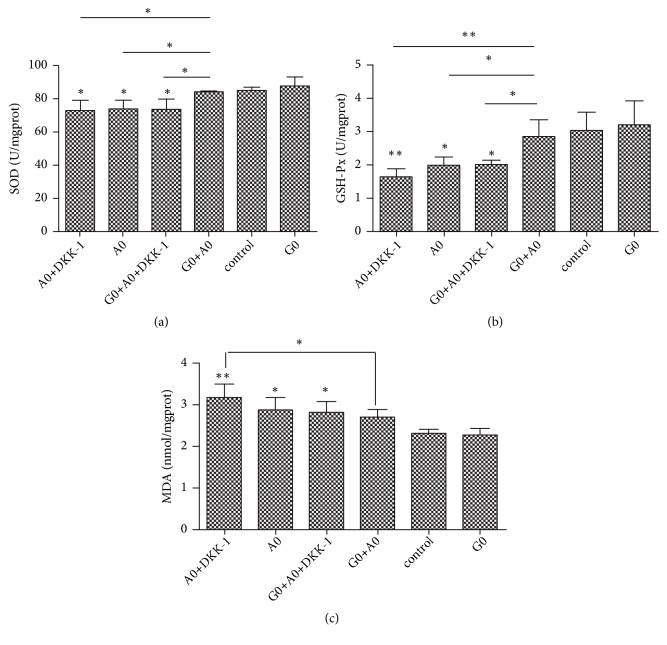
Glutamine strengthens the antioxidant capacity through activating the Wnt3a/*β*-catenin signaling pathway in vitro. (a) SOD activity in each group. (b) GSH-XP activity in each group. (c) MDA content in each group. Data were obtained from three independent experiments. The results were presented as the mean ± standard deviation. ^*∗*^p < 0.05 and ^*∗∗*^p < 0.01.

To elucidate the role of Wn3a/*β*-catenin signaling pathway on glutamine protecting PC12 cell against oxidative stress, DKK-1, Wn3a/*β*-catenin signaling pathway inhibitor was used. As shown in Figure 7, the activity of SOD and GSH-XP in A0+DKK-1, A0, and G0+A0+DKK-1 groups was significantly decreased compared with the control group, where the content of MDA was markedly increased, indicating that the antioxidant capacity was inhibited in AD cell model. Compared with the A0 group, the activity of SOD and GSH-XP in G0+A0 group was significantly increased, indicating that glutamine could improve antioxidant capacity of AD cell model. Compared with the G0+A0 group, the activity of SOD and GSH-XP in A0+DKK-1 and G0+A0+DKK-1 groups was significantly decreased, where the content of MDA was markedly increased, indicating that the glutamine strengthens the antioxidant capacity through activating the Wnt3a/*β*-catenin signaling pathway in vitro.

## 4. Discussion

Alzheimer disease (AD) is a multifactorial and fatal neurodegenerative disorder which has an influence on a large number of senior citizens. The neuropathological hallmarks of AD are A*β* plaques, neurofibrillary tangles, synapse loss, and neuronal loss [17, 18]. The pathogenesis and progression of AD are related to many risk factors, and the oxidative damage was served as the earliest pathological events [19]. The present study aimed to investigate the effect of glutamine on oxidative stress-induced injury in AD mice. Our results indicated that glutamine could protect against oxidative stress-induced injury in AD mice through activating the Wnt3a/*β*-catenin signaling pathway.

Initially, we performed the rotation experiment and found that the SAMP8 mice had an obvious rotational behavior. Following the administration of glutamine, the learning and memory abilities of SAMP8 mice were significantly increased. Concerning H&E and TUNEL staining experiments, we found that the hippocampus neuronal cells were abundant, arranged in neat rows, with morphological integrity and zonal distribution in SAMP8 mice following glutamine administration. And the administration of glutamine also prevented hippocampus neuronal cells from apoptosis. These results indicated that the glutamine could alleviate the damage of hippocampus. Glutamine, a free amino acid, confers various biological effects. The value of glutamine is particularly apparent during stress, which is extensively applied for the treatment of diseases related to inflammation and oxidative stress [20, 21]. Previous studies have showed that the glutamine levels are lower in the brains of patients with Alzheimer's disease (AD), and the glutamine supplementation could reduce inflammation-induced neuronal cell cycle activation, tau phosphorylation, and ATM-activation in a mouse model of AD [10]. Therefore, we speculated that the glutamine could reduce oxidative stress levels in hippocampus of AD mice to control AD progression. Superoxide dismutase (SOD) and glutathione peroxidase (GSH-XP) are important antioxidant enzymes in vivo, which are related to free radical scavenging ability. MDA is a lipid peroxidation product, and its content can reflect the level of free radicals [22]. At present study, we found that the glutamine restored the decrease of SOD and GSH-XP activity and the increase of MDA content in SAMP8 mice, indicating that the glutamine could improve antioxidant capacity of SAMP8 mice. Wnt3a/*β*-catenin signaling cascade was the common final pathway for neuroprotection and self-repair through antioxidative stress [23]. Wang Y L et al reported that the curcumin reduced oxidative stress-induced injury through activating Wnt3a/*β*-catenin signaling pathway in PD rats [24]. At present study, the Gln-high group exhibited significantly higher protein expression of Wnt3a and *β*-catenin as well as enhanced activity of SOD and GSH-XP and reduced MDA content, indicating that the glutamine may improve antioxidant capacity through activating Wnt3a/*β*-catenin signaling pathway in SAMP8 mice.

Additionally, we found that A*β*25-35 could cause injury to PC12 cells, while glutamine could provide protective effects against damage to PC12 cells. As a widely used neurotoxin in the construction of AD models, A*β*25-35 increases neuronal loss, inflammation, oxidative stress, and cognitive and memory impairment [25]. Our study found that the glutamine treatment increased SOD and GSH-XP activity and decreased MDA content. The expression of Wnt3a and *β*-catenin protein was significantly increased following glutamine treatment. Interestingly, when the Wnt3a/*β*-catenin signaling cascade was inhibited by DKK-1, SOD, and GSH-Px activity was reduced, while MDA content was increased. These results indicated that the glutamine exerts its antioxidant capacity by activating the Wnt3a/*β*-catenin signaling pathway.

In summary, our results demonstrated that glutamine plays a protective role against oxidative stress-related injury in SAMP8 mice and PC12 cells through activating the Wnt3a/*β*-catenin signaling pathway to enhance viability and attenuate apoptosis. Our study provides potential therapeutic strategies in the future treatment of oxidative stress-related injury involved in AD as well as the underlying mechanism of the neuroprotection function of glutamine. Considering the complexity of molecular and neurological systems, further efforts are needed to confirm our findings.

## Figures and Tables

**Table 1:** Glutamine affects the learning and memory abilities of SAMP8 mice.

Group	Learning ability		Memory ability	
	Reaction time (s)	Error time (s)	Reaction time (s)	Error time (s)
Control (n=10)	16.58±1.62	3.84±1.08	197.40±24.05	3.54±0.97
Model (n=10)	64.69±5.55*∗∗*	6.93±1.82*∗∗*	71.32±8.12*∗∗*	7.07±1.40*∗∗*
Gln-low (n=10)	60.01±5.32*∗∗*	6.82±2.14*∗∗*	76.22±6.70*∗∗*	6.52±1.71*∗∗*
Gln-high (n=10)	18.91±2.19^##^	4.21±1.09^#^	182.26±15.79^##^	4.32±1.46^##^

^*∗∗*^p < 0.01, compared with control group. ^#^p < 0.05 and ^##^p < 0.01, compared with model group.

## Data Availability

The data used to support the findings of this study are available from the corresponding author upon request.
